# Editorial: Gambling during COVID-19: Changes, risks, challenges and opportunities in the wake of COVID-19

**DOI:** 10.3389/fpsyg.2022.1110963

**Published:** 2023-01-04

**Authors:** David Forsström, Philip Lindner, Tobias Hayer

**Affiliations:** ^1^Department of Psychology, Uppsala University, Stockholm, Sweden; ^2^Department of Clinical Neuroscience, Centre for Psychiatry Research, Stockholm Health Care Services, Karolinska Institutet, Stockholm, Sweden; ^3^Department of Psychology, Stockholm University, Stockholm, Sweden; ^4^Centre for Dependency Disorders, Stockholm Health Care Services, Stockholm, Sweden; ^5^Department for Health and Society, Institute of Public Health and Nursing Research, University of Bremen, Bremen, Germany

**Keywords:** COVID-19, gambling, effects, risk, changes

To date, the COVID-19 pandemic has resulted in an estimated 6.61 million deaths from a total of 636 million infected worldwide. With no vaccines available at the start of the pandemic, many countries resorted to imposing quarantine procedures, restrictions, and other measures designed to encourage and enforce social distancing. The short- and long-term effects of these measures on mental health and addictive behaviors are still not fully understood. However, the impact of COVID-19 on gambling, both a popular leisure activity around the world and a potential addictive behavior, was quickly put in the scientific spotlight due to changed betting opportunities, closed casinos, a perceived increased need for coping with mental health problems, financial burdens, and more. This, in turn, even prompted changed legislation and policies in several jurisdictions. The field of gambling consists, on the one hand, of a wide range of gambling products and, on the other hand, of many different sub-groups with varying gambling motives. These heterogeneities pose a challenge to researchers examining the full consequences of the pandemic on gambling.

There is growing consensus in the field that total gambling activity decreased during the early stages of the pandemic, largely fueled by the cancellation of sporting events and closures of land-based gambling options. However, research has also revealed that some vulnerable groups increased their gambling activities. These groups were formed by individuals of younger age, men, and individuals with a history of problem gambling (Brodeur et al., 2021; Hodgins and Stevens, 2021; Quinn et al., 2022). So far, this appears to hold true for all the different waves of the pandemic.

Despite the available studies and reviews, there is still a need to explore different aspects of how COVID-19 has affected gambling, the prevention of gambling problems, and the treatment of gambling disorders. Considering this, the focus of this Research Topic was to examine the impact of COVID-19 on gambling from a broad perspective, bringing together a variety of research disciplines, methods, and data sources to provide a comprehensive picture with evidence-based policy implications. In total, eight papers were included. A position paper (Håkansson et al.) addressed the risks of match fixing as a consequence of the pandemic and called for more research in this area. The pandemic, with the cessation of sporting events, may have put athletes more at risk of developing mental illness and of increasing their gambling, which might in turn have resulted in a higher propensity of engaging in match fixing. The paper, thus, called for more research focused on risks and consequences of match fixing, longitudinal data on this issue, and prevention and treatment of problem gambling among athletes. Another study (Baenas et al.) focused on adherence to treatment in patients with gambling disorders before and during the pandemic. Treatment duration (i.e., shorter treatment periods) was a predictor for drop out from treatment both pre- and post-lockdown. Further, lower levels of anxiety and depressive symptoms due to the pandemic and higher socio-economic positions predicted dropout rates during the lockdown. Finally, employment concerns related to the pandemic were associated with post-lockdown dropout. A qualitative study investigated the closure of places to gamble on electronic gambling machines (EGMs) in Finland (Marionneau and Järvinen-Tassopoulos). The results suggested that many individuals in the sample were happy that the EGMs were closed and wished that the machines stayed closed. Furthermore, for many of the participants, the closure meant that they stopped gambling and did not substitute their activities with other types of gambling. However, some participants migrated to high-risk online gambling games. From a policy perspective, limiting access to EGMs in Finland and the need for more responsible gambling features online to limit harm represent two implications of this study. A similar study with quantitative data investigated the closure of land-based venues in Germany (Kalke et al.). Almost half of the respondents ceased to gamble during the first lockdown. More specifically, women, younger individuals, and individuals with lower gambling frequency were more like to cease gambling. Interestingly, only a small minority of study participants switched to online gambling. Finally, individuals with more pronounced cognitive distortions switched to online gambling more often. An Italian study investigated the state of affected family members in relation to the pandemic (Donati et al.). The results of this small-scale study showed that family members were in a more positive emotional state due to closures of offline gambling opportunities, but they still faced problems in their relationships with people with gambling disorders. The authors concluded that affected family members are at risk of developing other problematic behaviors, such as excessive television watching. Two other studies investigated longitudinal changes in the level of gambling in the population (for Hungary: Koós et al.; for Sweden: Månsson et al.). The Hungarian study investigated different types of potentially addictive behaviors, such as problematic social media use, Internet gaming disorder, gambling disorder, problematic pornography use, and compulsive sexual behavior disorder. The study found no significant increase of these behaviors during the pandemic. The same holds true for the Swedish study that explored gambling during the first and second wave of the pandemic (i.e., no increase in gambling activities). Adding to this, there was no shift from sports betting to other types of gambling. However, in Sweden, increased gambling problems from the first to the second wave were predicted to be caused by gambling on high-risk gambling forms and worrying about mental health in relation to the pandemic. Finally, another study from Sweden examined gambling in a psychiatric sample (Forsström et al.). The study found a high prevalence of problem and at-risk gambling among the included participants both for those who gambled before the pandemic and those who started during the pandemic. Higher degrees of worry, isolation, and psychiatric disorders were associated with higher degrees of problem gambling among those who started gambling during the pandemic. Important to note is that worries associated with COVID-19 predicted gambling problems.

In summary, the included studies highlighted the multitude of issues that gamblers, significant others, and the industry face in the post-pandemic era. Included studies found that different types of pandemic-based burdens were linked to increases in gambling or shifts to other forms of gambling, indicating that, in order to prevent harm, there is a need to monitor these aspects among individuals that gamble. With the global distribution of effective vaccines, the immediate dangers from COVID-19 have now largely subsided and restrictions have been lifted in most parts of the world, where everyday life has returned to normal levels. However, with a potential global recession looming and the threat of new pandemics hitting a vulnerable world, it is important that policymakers learn as much as possible about the full immediate and long-term public health effects of the COVID-19 pandemic. We hope that the studies included in this Research Topic will provide some valuable implications for future research activities in the field of gambling.

## Author contributions

DF: conceptualization, writing—original draft, and writing—review and editing. PL and TH: validation and writing—review and editing. All authors contributed to the article and approved the submitted version.
